# Supplementary figures and images for: Mitochondrial DNA is a sensitive surrogate and oxidative stress target in oral cancer cells
Source: PLoS One. 2024 Sep 3;19(9):e0304939. doi: 10.1371/journal.pone.0304939 (PMC11371132; doi:10.1371/journal.pone.0304939)

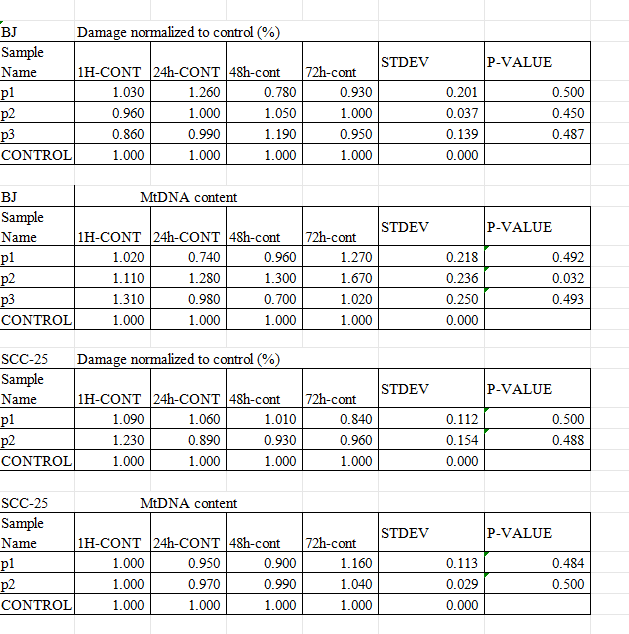

Supplement: S1 Fig — The values behind the means, standard deviations and the values used to build graphs, the points extracted from images for analysis, all include in these figures. (ZIP) [file pone.0304939.s001.zip › S1_Fig1-original data/S1_Fig1-1original data.tif.png]

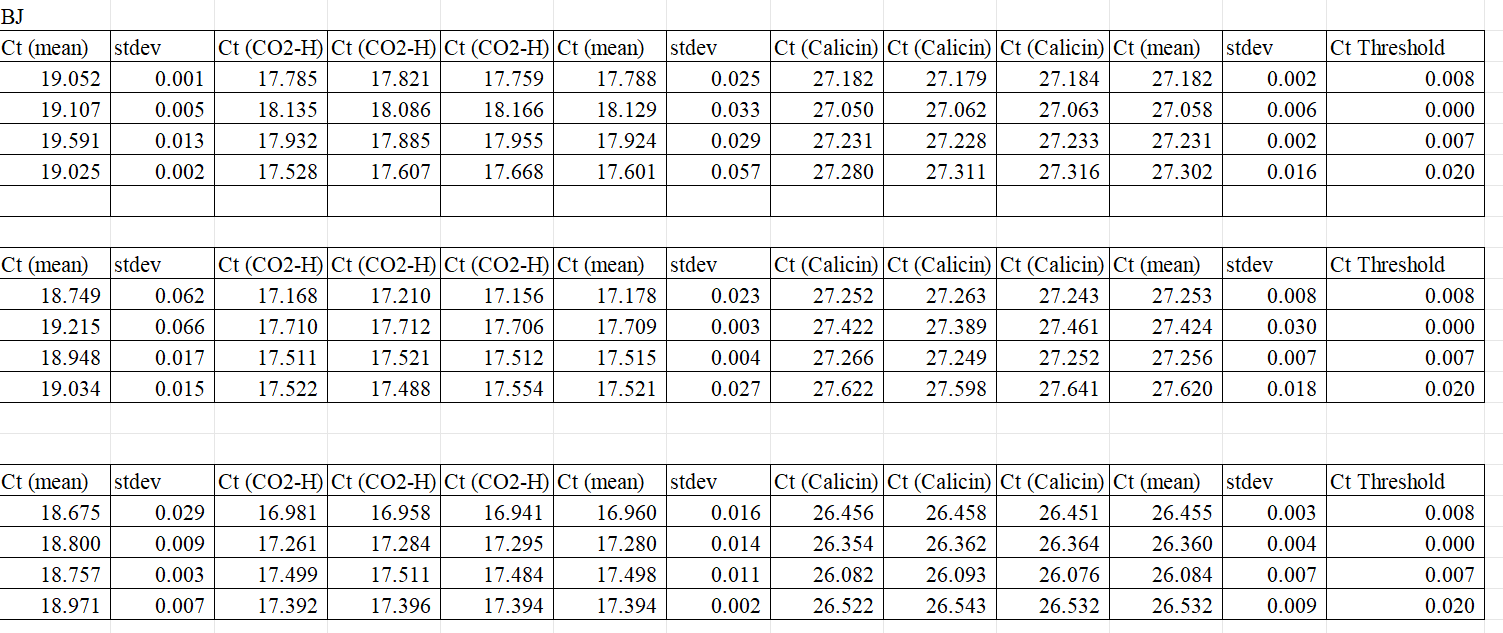

Supplement: S1 Fig — The values behind the means, standard deviations and the values used to build graphs, the points extracted from images for analysis, all include in these figures. (ZIP) [file pone.0304939.s001.zip › S1_Fig1-original data/S1_Fig1-2 original data.tif.png]

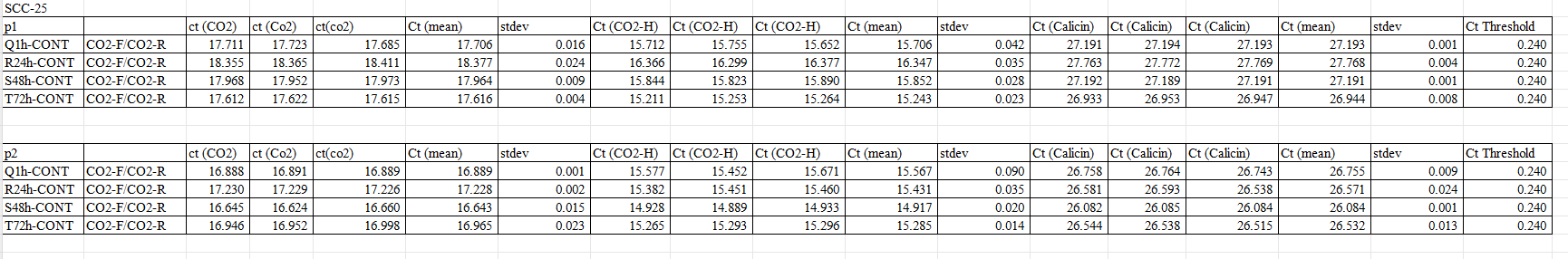

Supplement: S1 Fig — The values behind the means, standard deviations and the values used to build graphs, the points extracted from images for analysis, all include in these figures. (ZIP) [file pone.0304939.s001.zip › S1_Fig1-original data/S1_Fig1-3 original data.tif.png]

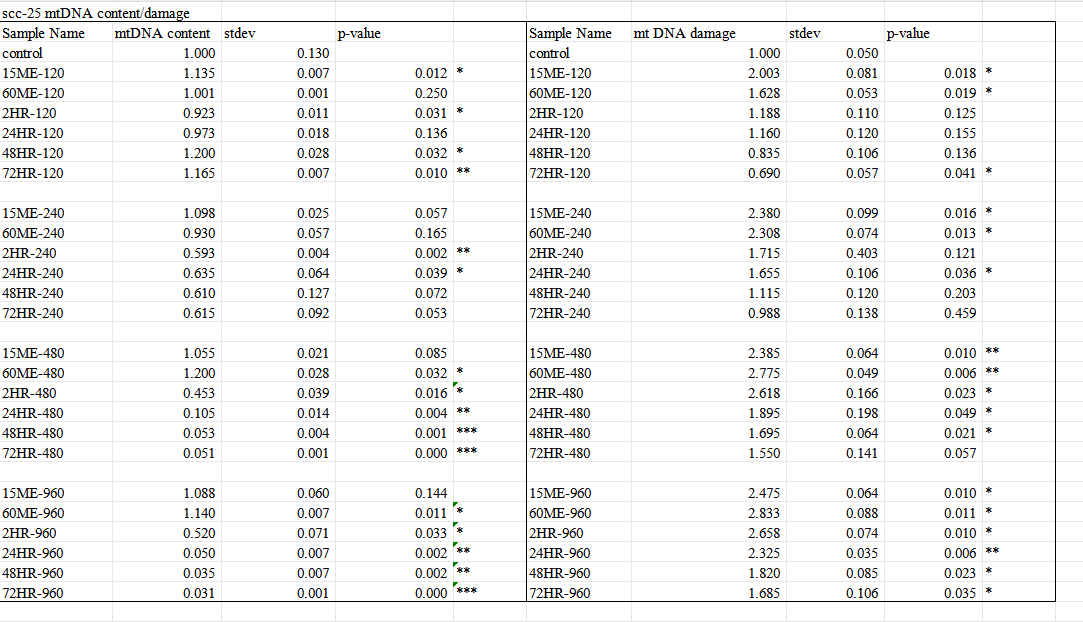

Supplement: S2 Fig — The values behind the means, standard deviations and the values used to build graphs, the points extracted from images for analysis, all include in these figures. (ZIP) [file pone.0304939.s002.zip › S2_Fig2-original data/S2_Fig2-1 -1original data.tif.png]

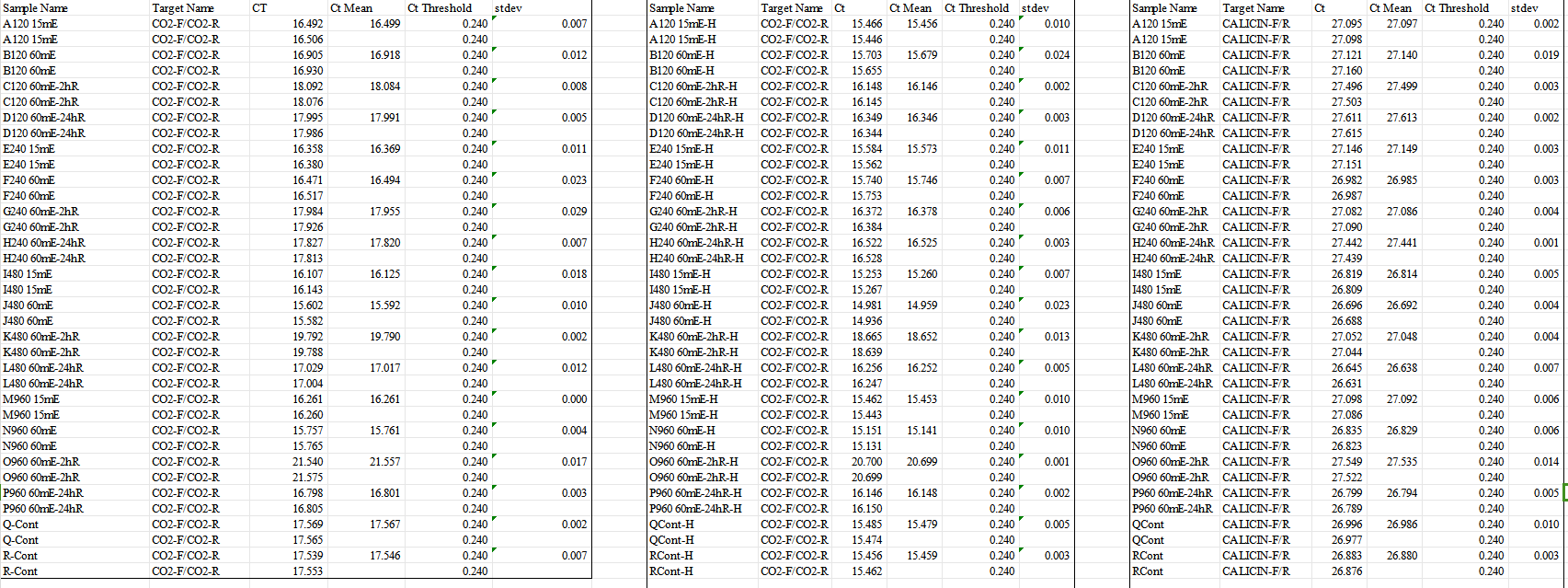

Supplement: S2 Fig — The values behind the means, standard deviations and the values used to build graphs, the points extracted from images for analysis, all include in these figures. (ZIP) [file pone.0304939.s002.zip › S2_Fig2-original data/S2_Fig2-1-2 original data.tif.png]

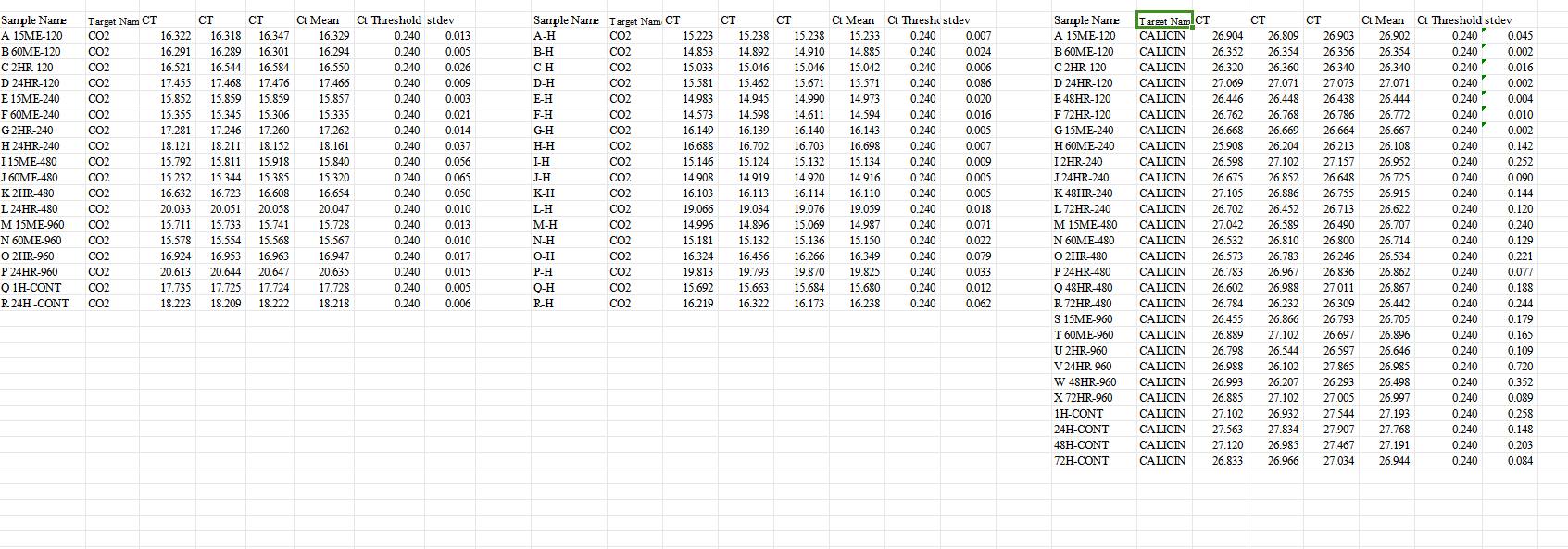

Supplement: S2 Fig — The values behind the means, standard deviations and the values used to build graphs, the points extracted from images for analysis, all include in these figures. (ZIP) [file pone.0304939.s002.zip › S2_Fig2-original data/S2_Fig2-1-3 original data.tif.png]

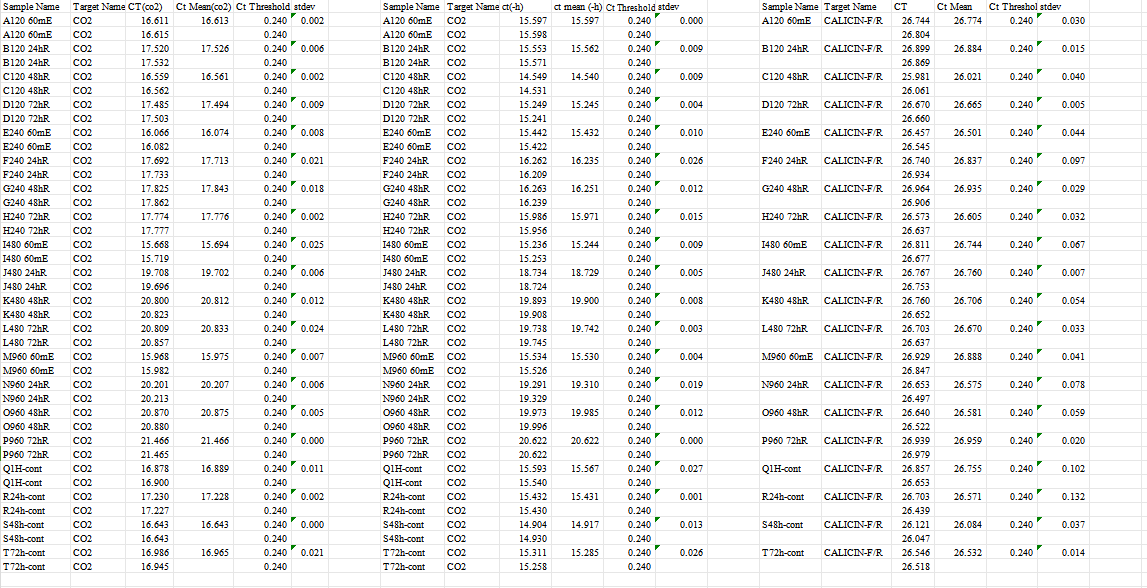

Supplement: S2 Fig — The values behind the means, standard deviations and the values used to build graphs, the points extracted from images for analysis, all include in these figures. (ZIP) [file pone.0304939.s002.zip › S2_Fig2-original data/S2_Fig2-1-4 original data.tif.png]

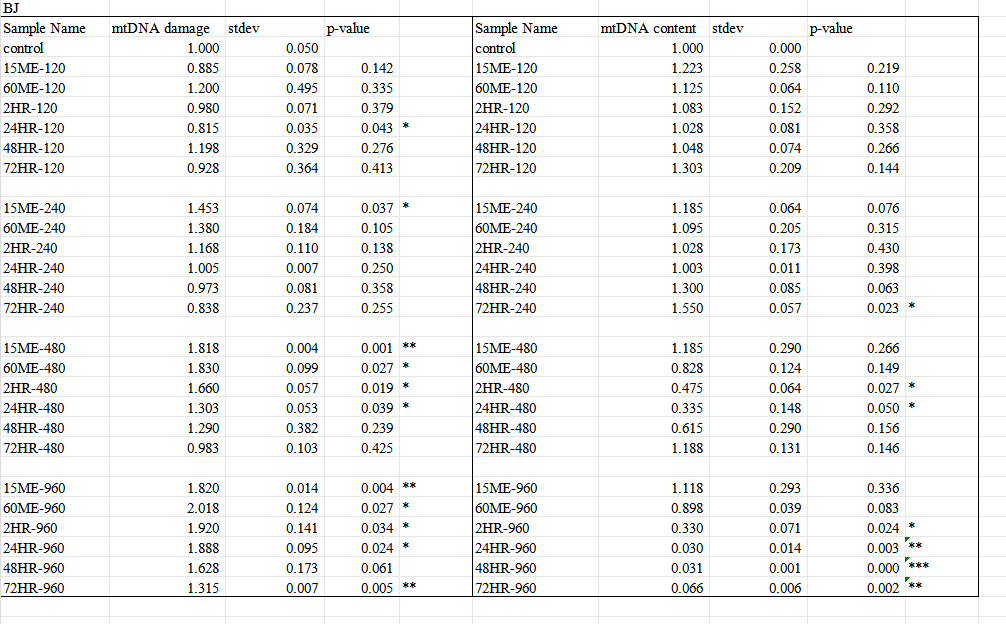

Supplement: S2 Fig — The values behind the means, standard deviations and the values used to build graphs, the points extracted from images for analysis, all include in these figures. (ZIP) [file pone.0304939.s002.zip › S2_Fig2-original data/S2_Fig2-2-1original data.tif.png]

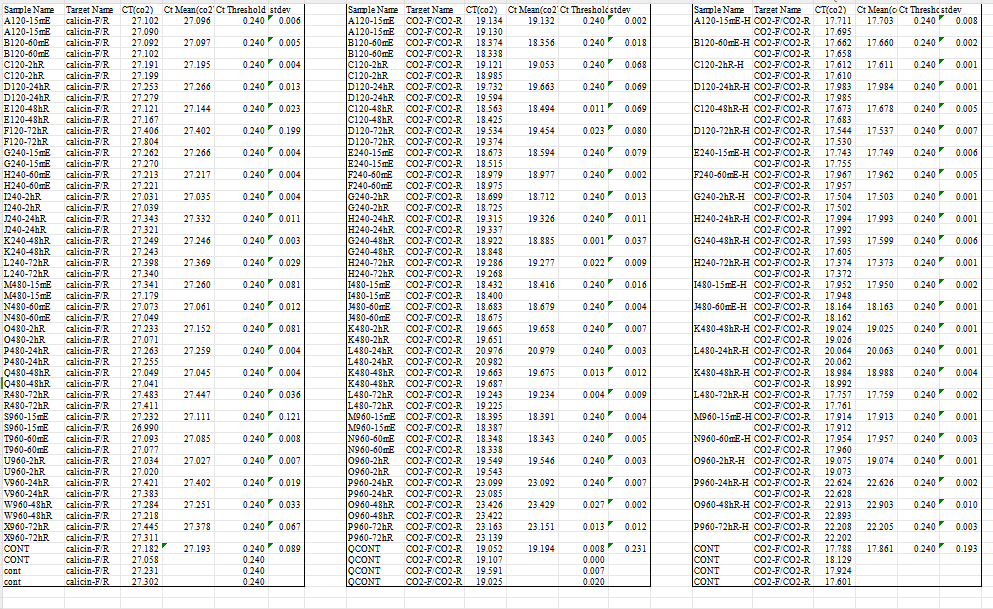

Supplement: S2 Fig — The values behind the means, standard deviations and the values used to build graphs, the points extracted from images for analysis, all include in these figures. (ZIP) [file pone.0304939.s002.zip › S2_Fig2-original data/S2_Fig2-2-2 original data.tif.png]

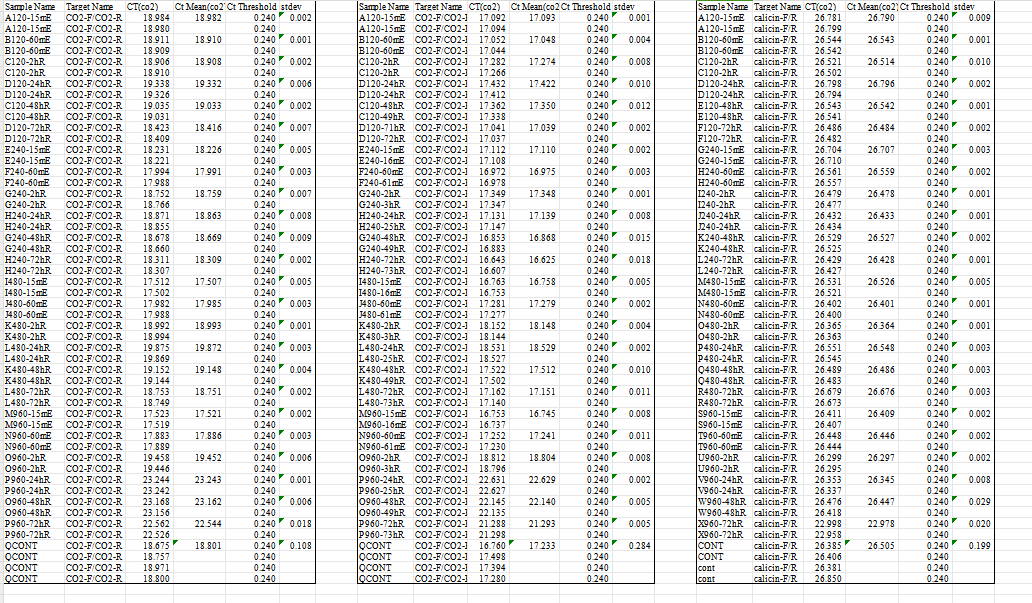

Supplement: S2 Fig — The values behind the means, standard deviations and the values used to build graphs, the points extracted from images for analysis, all include in these figures. (ZIP) [file pone.0304939.s002.zip › S2_Fig2-original data/S2_Fig2-2-3 original data.tif.png]

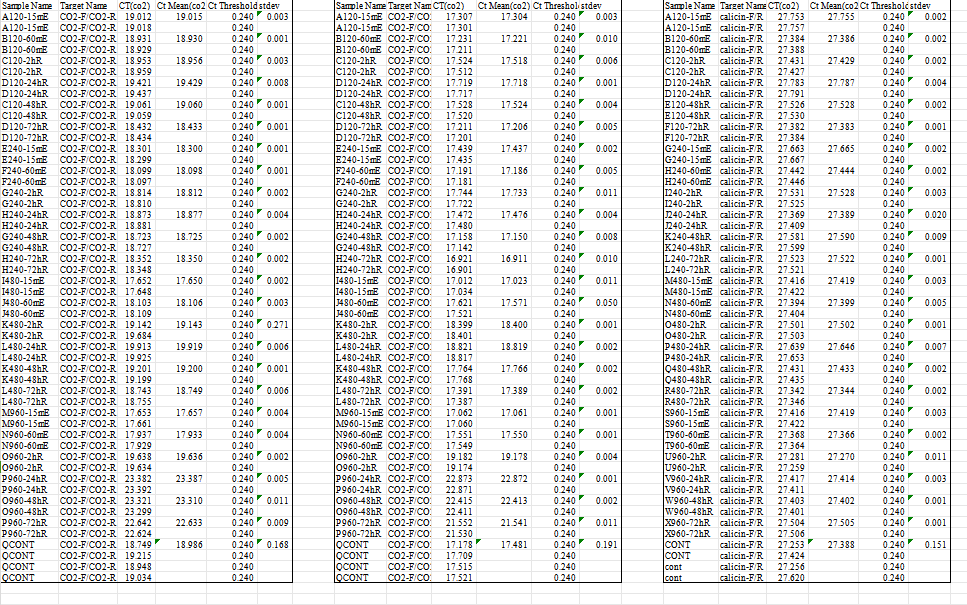

Supplement: S2 Fig — The values behind the means, standard deviations and the values used to build graphs, the points extracted from images for analysis, all include in these figures. (ZIP) [file pone.0304939.s002.zip › S2_Fig2-original data/S2_Fig2-2-4 original data.tif.png]

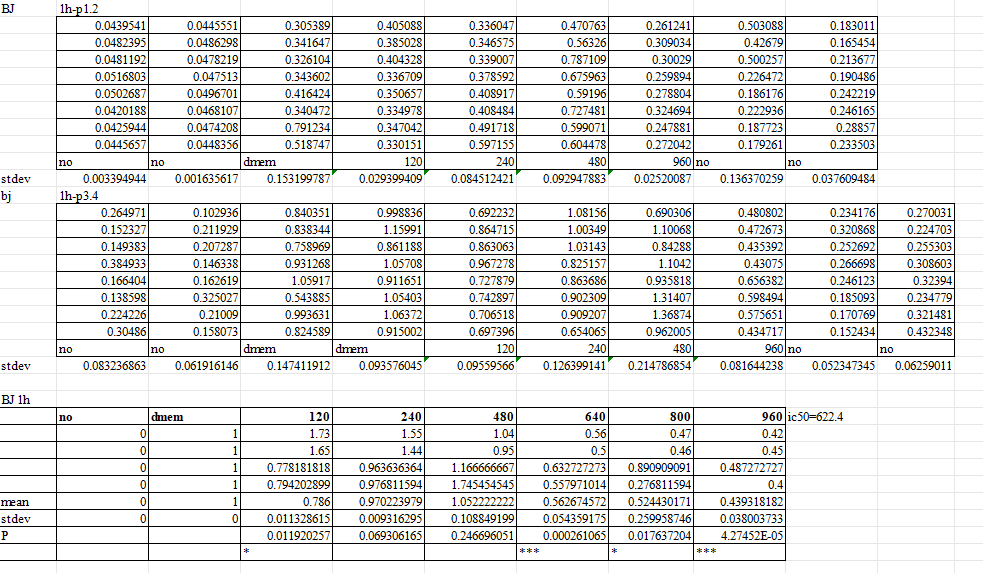

Supplement: S3 Fig — The values behind the means, standard deviations and the values used to build graphs, the points extracted from images for analysis, all include in these figures. (ZIP) [file pone.0304939.s003.zip › S3_Fig3-original data/S3_Fig3-1original data.tif.png]

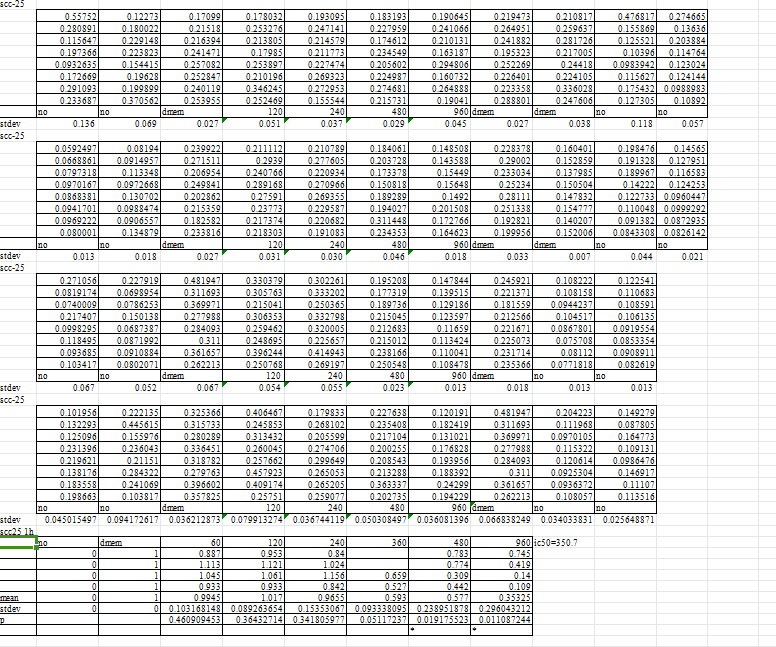

Supplement: S3 Fig — The values behind the means, standard deviations and the values used to build graphs, the points extracted from images for analysis, all include in these figures. (ZIP) [file pone.0304939.s003.zip › S3_Fig3-original data/S3_Fig3-2original data.tif.png]

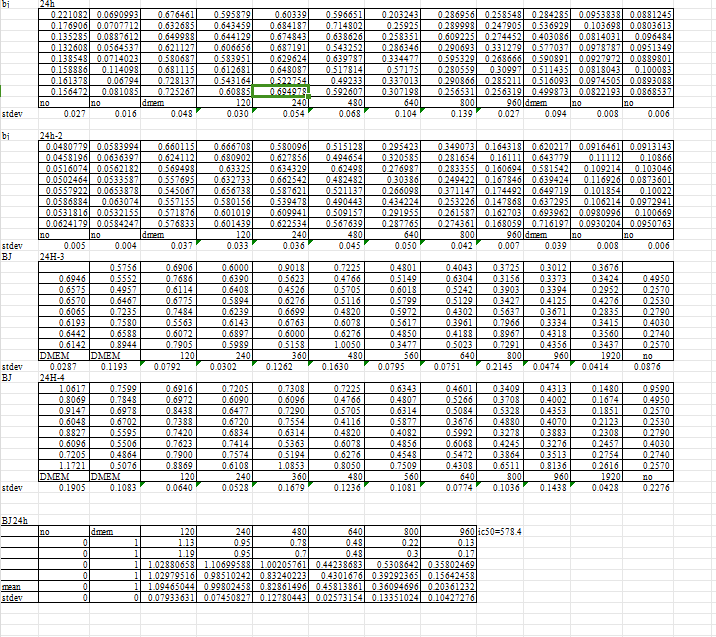

Supplement: S3 Fig — The values behind the means, standard deviations and the values used to build graphs, the points extracted from images for analysis, all include in these figures. (ZIP) [file pone.0304939.s003.zip › S3_Fig3-original data/S3_Fig3-3original data.tif.png]

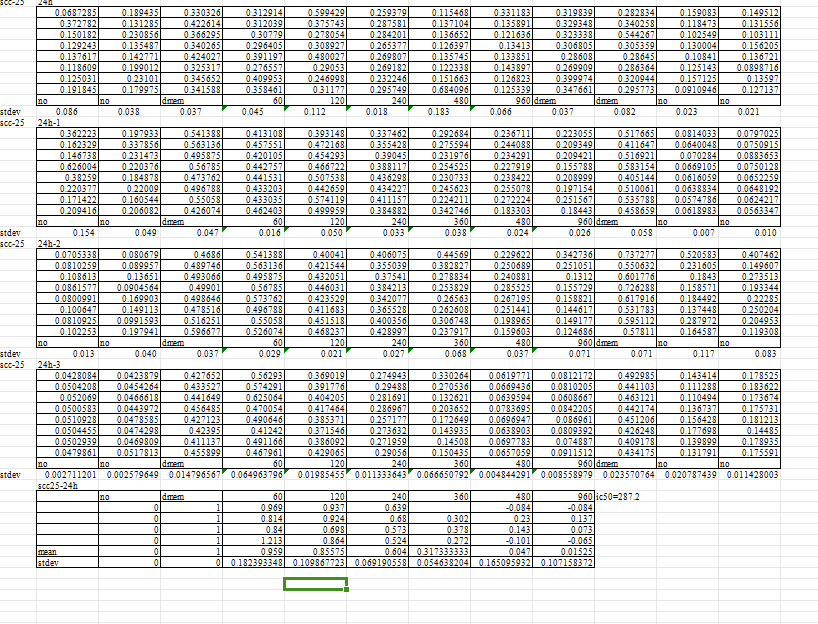

Supplement: S3 Fig — The values behind the means, standard deviations and the values used to build graphs, the points extracted from images for analysis, all include in these figures. (ZIP) [file pone.0304939.s003.zip › S3_Fig3-original data/S3_Fig3-4 original data.tif.png]

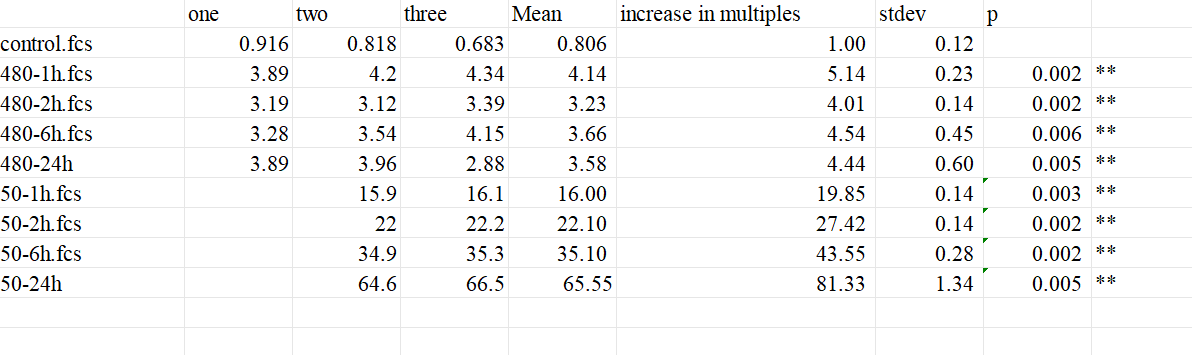

Supplement: S4 Fig — The values behind the means, standard deviations and the values used to build graphs, the points extracted from images for analysis, all include in these figures. (ZIP) [file pone.0304939.s004.zip › S4_Fig4-original data/S4_Fig4 original data.tif.png]

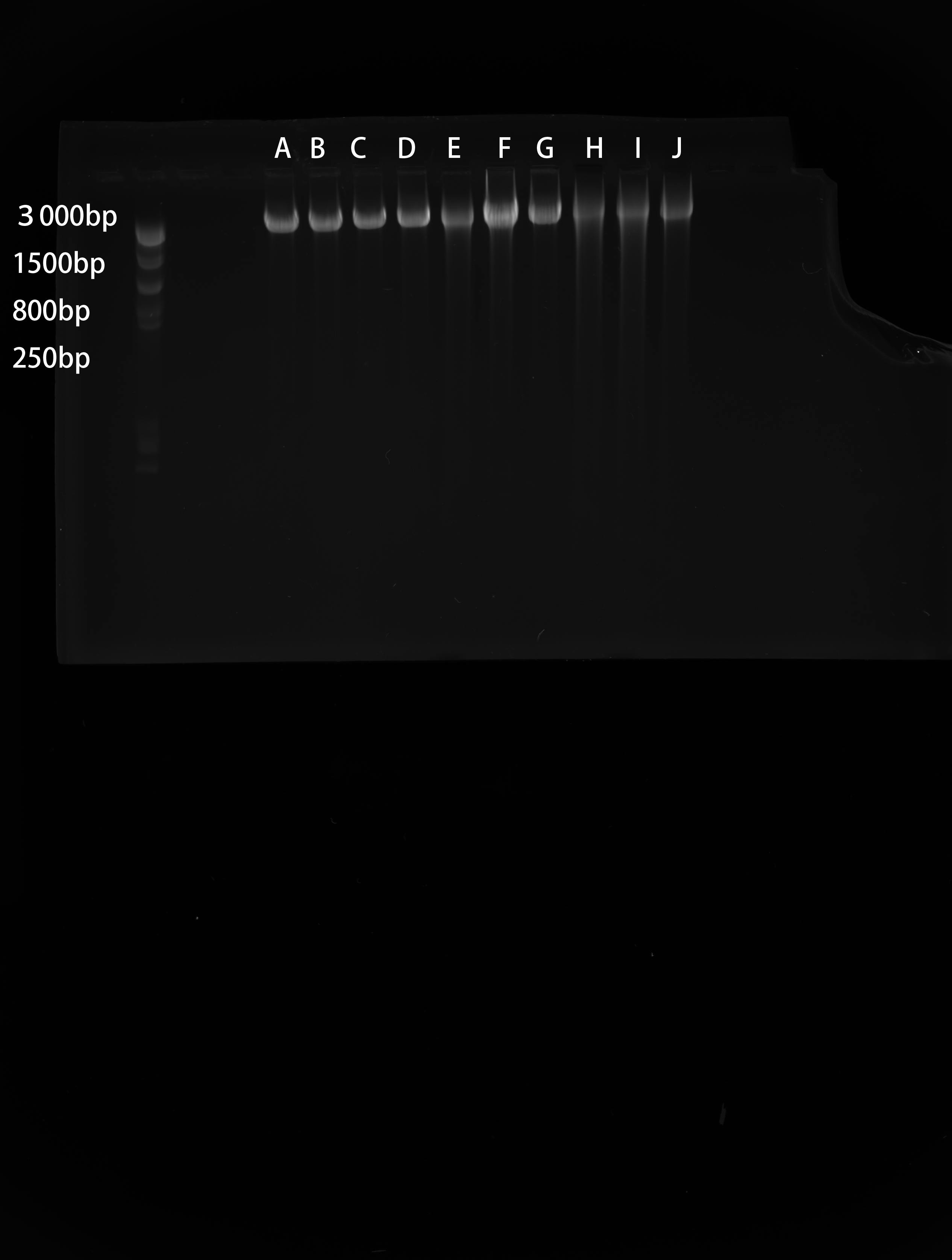

Supplement: S5 Fig — The original gel electrophoresis images are included, along with the specific sample names and sizes represented by each band. (ZIP) [file pone.0304939.s005.zip › S5_Fig5-original data/S5_Fig5 original data.tif.tif]

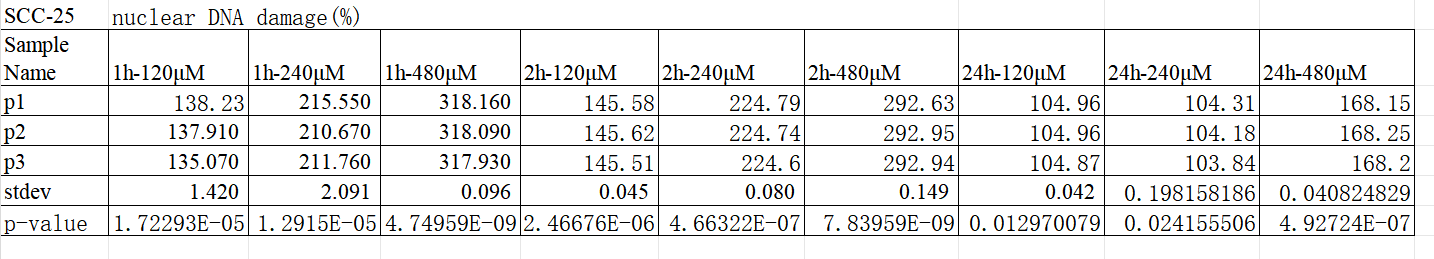

Supplement: S6 Fig — The values behind the means, standard deviations and the values used to build graphs, the points extracted from images for analysis, all include in these figures. (ZIP) [file pone.0304939.s006.zip › S6_Fig6-original data/S6_Fig6original data.tif.tif]
