# Supplementary material for: Mitochondrial DNA is a sensitive surrogate and oxidative stress target in oral cancer cells
Source: PLoS One. 2024 Sep 3;19(9):e0304939. doi: 10.1371/journal.pone.0304939 (PMC11371132; doi:10.1371/journal.pone.0304939)
Supplement: S5 Fig — The original gel electrophoresis images are included, along with the specific sample names and sizes represented by each band. (ZIP) [file pone.0304939.s005.zip › S5_Fig5-original data/S5_raw_images notes.pdf]

S1_raw_images

1.Experimentation ： Single Stranded Break (SSB) detection in SCC-25 cells continuously exposed to 120/240/480 μM hydrogen peroxide (H_2O_2) for 1 h/2 h/24 h.

2.Image annotation： A - control;

B - 120 μM H_2O_2 exposure for 1 h;

C - 120 μM H_2O_2 exposure for 2 h;

D - 120 μM H_2O_2 exposure for 24 h;

E - 240 μM H_2O_2 exposure for 1 h;

F- 240 μM H_2O_2 exposure for 2 h;

G - 240 μM H_2O_2 exposure for 24 h;

H - 480 μM H_2O_2 exposure for 1 h;

I - 480 μM H_2O_2 exposure for 2 h;

J - 480 μM H_2O_2 exposure for 24 h.

3.Description of image cropping in the original text：The gel images used in the manuscript are unedited raw images, presented in their entirety in the article without any modifications.
